# A Case Report on a Hybrid Approach to Managing Acute Large Bowel Obstruction Secondary to Spigelian Hernia

**DOI:** 10.7759/cureus.53869

**Published:** 2024-02-08

**Authors:** Isabelle Huynh, Wei Mou Lim, Michelle Zhiyun Chen, Senthilkumar Rajavel Sundaramurthy, Yeng Kwang Tay

**Affiliations:** 1 General Surgery and Acute Surgery Unit, Monash Health, Melbourne, AUS; 2 Colorectal Unit, Monash Health, Melbourne, AUS

**Keywords:** general and colorectal surgeon, emergency abdominal surgery, exploratory laparoscopy, large-bowel obstruction, rare case of spigelian hernia

## Abstract

Spigelian hernias are an uncommon type of primary ventral hernia and are defined as a defect in the Spigelian aponeurosis (fascia). Herein, we present an uncommon case of Spigelian hernia to highlight the potential complications of these hernias and the need for surgical management. This is a case report of an 86-year-old gentleman presenting post-fall with an acute rib fracture and an incidental Spigelian hernia seen on a CT trauma pan scan. The Spigelian hernia surgical treatment was planned for elective management due to the anesthetic risks associated with an elderly patient and acute rib fractures. Ultimately, the patient developed a large bowel obstruction secondary to the Spigelian hernia and required emergency operative management to relieve the obstruction. The patient had an uncomplicated recovery following his emergency surgery.

This case report highlights the importance of assessing anesthetic risks versus surgical risks when it comes to surgical planning. Clinicians should recognize occult hernias and continue ongoing clinical reviews with a high index of suspicion, as symptoms of Spigelian hernia obstruction might be non-specific.

## Introduction

A Spigelian hernia is a rare ventral hernia that is defined as herniation of abdominal contents or peritoneum through a defect, namely, the Spigelian fascia, which consists of the transversus abdominis and the internal oblique aponeuroses [1]. These hernias are an uncommon type of primary ventral hernia with an incidence ranging from 0.12% to 2% of all abdominal wall hernias [1]. Predisposing factors include female gender and those over 60 years of age [2]. Once diagnosed, they require urgent repair due to high rates of complications of strangulation and bowel obstruction. This case report combines the delicate balance between anesthetic and surgical complications in emergency surgical management. The risks posed for this patient were related to their initial presentation of a fall with rib fractures. This was a consideration as pulmonary complications account for 10-40% of postoperative complications after abdominal and vascular surgery [3].

Numerous techniques have been proposed for repairing Spigelian hernias and there is no gold standard for operative management. Herein, we discuss our management of a rare case of Spigelian hernia and large bowel obstruction using a hybrid technique. This case report has been reported in line with the consensus Surgical CAse REport (SCARE) criteria [4].

## Case presentation

**Figure 1:**
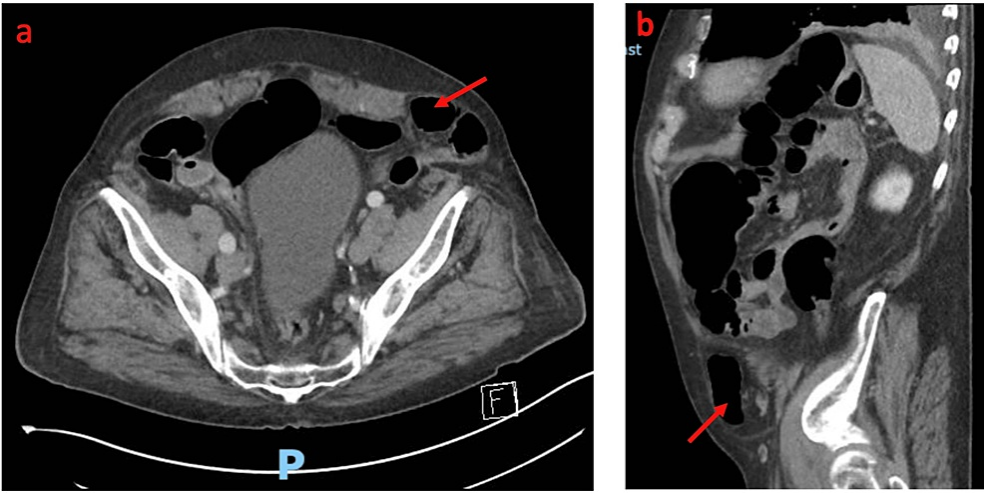
a and b: Axial and sagittal CT abdomen and pelvis with IV contrast showing a sigmoid colon in a left spigelian hernia (red arrows)

An 86-year-old gentleman presented to the emergency department due to an unwitnessed fall at home. CT imaging showed a displaced eighth posterior left rib fracture and an incidental finding of a left Spigelian hernia, containing a loop of sigmoid colon, without evidence of large bowel obstruction (Figures 1a, 1b). The patient was admitted to the medical unit for observation and analgesia, with a planned elective repair of his Spigelian hernia.

**Figure 2:**
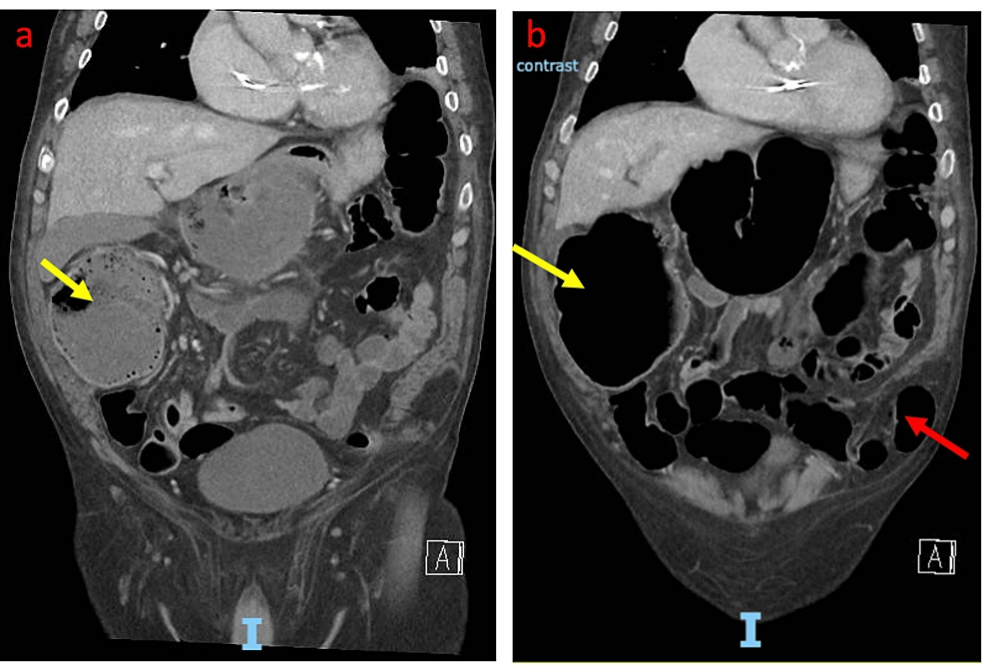
a and b: Coronal CT abdomen and pelvis with IV contrast confirming a large bowel obstruction with cecal dilatation, pneumatosis (yellow arrow), and Spigelian hernia containing the sigmoid (red arrow)

Three days post-admission, the patient developed abdominal pain, distension, and obstipation. An abdominal X-ray showed markedly distended large bowel loops. Repeat CT abdomen and pelvis demonstrated cecal pneumatosis and a large bowel obstruction with a transition point at the site of the left Spigelian hernia containing the sigmoid colon (Figures 2a, 2b). The hernial defect size was two centimeters.

The patient underwent an emergency open Spigelian hernia repair, followed by colonoscopic decompression of the colon, on-table laparoscopy to confirm a complete reduction of the hernia, and an assessment of the serosa of the colon. The cecum had no signs of ischemia on the serosa and luminal inspection. Postoperatively the patient was monitored for bowel activity, adequate pain control, potential respiratory compromise, and general physical function. The patient had an uncomplicated recovery and was discharged following medical optimization including adequate post-operative analgesia and physiotherapy, 12 days post-surgery. He was followed up in our post-surgical clinic two months post-operation with no further surgical follow-up required thereafter.

## Discussion

Spigelian hernias are defined as a defect in the Spigelian aponeurosis (fascia) that consists of the transversus abdominis and internal oblique aponeuroses. Spigelian hernias can occur anywhere along the Spigelian aponeurosis but they are most commonly found in the region from just below the level of the umbilicus to the interspinal plane, commonly referred to as the Spigelian hernia belt [1]. When asymptomatic, it may be an incidental finding, diagnosed on an imaging test or during abdominal surgery for another reason [5].

At present, there are several published case reports on colonic obstruction associated with a Spigelian hernia. The majority of them were repaired using an open approach due to the emergent nature of their presentation. High fever, tachycardia, and peritonitis suggest strangulation, perforation, or ischemia and usually require an urgent laparotomy [6]. However, this precludes the assessment of other parts of the colon, especially in this case, where there was evidence of cecal pneumatosis on imaging. Minimally invasive techniques utilizing laparoscopy in highly selected stable patients allow assessment of the entire colon. It offers a clear view of the entire fascial defect, which is not always possible with the open technique [7]. Direct localization on the abdominal wall can sometimes be difficult due to the hernia being obscured by the overlying external oblique aponeurosis. Our patient was surgically managed with a hybrid technique: open reduction and repair of the Spigelian hernia, endoscopic decompression of the colon as well as assessment of the mucosa for evidence of ischemia, and laparoscopic assessment of the serosa of the large bowel and hernia defect.

The conundrum with proceeding to emergent repair at presentation lies with the acute rib fracture and the risk of respiratory and anesthetic complications associated with surgery. The patient was managed conservatively with watchful waiting and daily clinical reviews. As soon as clinical deterioration was detected, clinical assessment and radiological images were employed. It was then agreed between the surgical and anesthetic teams that surgical management outweighed the anesthetic-driven delay. Care was taken to observe the patient intraoperatively and postoperatively to ensure safe surgical recovery.

Currently, there is no consensus on the gold standard for the treatment of Spigelian hernia with a large bowel obstruction. A literature review on current approaches to the surgical treatment of Spigelian hernias concluded that all diagnosed Spigelian hernias should be planned for elective operation to prevent strangulated hernia [8]. Both open and laparoscopic Spigelian hernia treatment can be safely performed, depending on the surgeon’s experience. In most cases, a mesh repair is generally advised [9].

## Conclusions

Large bowel obstruction caused by a Spigelian hernia is an extremely rare entity. Spigelian hernias are clinically elusive often until strangulation occurs. Once diagnosed, an operation should always be advised. In cases where anesthetic complications may complicate surgery, the preferred option would be to delay management. Most Spigelian hernias are planned for early elective repair, however, there is a risk that surgical emergencies, such as large bowel obstruction, can occur if surgery is delayed.

It is important to educate clinicians and patients on symptoms relating to obstruction such as obstipation, abdominal pain, and distension. This case highlights the importance of ongoing clinical review and the importance of recognizing occult hernias with a high index of suspicion, as symptoms of Spigelian hernia obstruction might be non-specific.
